# The effects of artificial light at night on spider brains

**DOI:** 10.1098/rsbl.2024.0202

**Published:** 2024-09-04

**Authors:** Nikolas J. Willmott, Jay R. Black, Kathryn B. McNamara, Bob B. M. Wong, Therésa M. Jones

**Affiliations:** ^1^ School of BioSciences, The University of Melbourne, Melbourne, Victoria 3010, Australia; ^2^ School of Geography, Earth and Atmospheric Sciences, The University of Melbourne, Melbourne, Victoria 3010, Australia; ^3^ Trace Analysis for Chemical, Earth and Environmental Sciences (TrACEES) Platform, The University of Melbourne, Melbourne, Victoria 3010, Australia; ^4^ School of Biological Sciences, Monash University, Clayton, Victoria 3800, Australia

**Keywords:** anthropogenic light, neuroanatomy, araneidae, pollution, urban, plasticity

## Abstract

Artificial light at night (ALAN) is an increasingly pervasive pollutant that alters animal behaviour and physiology, with cascading impacts on development and survival. Recent evidence links exposure to ALAN with neural damage, potentially due to its action on melatonin synthesis, a powerful antioxidant. However, these data are scarce and taxonomically limited. Here, we used micro-CT to test the effects of short-term ALAN exposure on brain volumes in the Australian garden orb-weaving spider (*Hortophora biapicata*), a species commonly found in urban areas and, specifically, around street lights. We found that short-term ALAN exposure was linked to reductions in the volumes of brain structures in the primary eye visual pathway, potentially as a consequence of oxidative stress or plastic shifts in neural investment. Although the effects of ALAN were subtle, they provided new insights into potential mechanisms underpinning the behavioural and physiological impacts of ALAN in this important urban predator.

## 1. Introduction

Artificial light at night (ALAN) alters natural cycles of light [1,2] and is linked to the disruption of a multitude of light-dependent biological rhythms and physiological functions [3–7]. Light-related impacts differ drastically between taxa [8–10], but the mechanisms underpinning these differences remain poorly understood. Recent evidence, largely from vertebrates, suggests that biological impacts of ALAN can be driven by effects on the brain, through either light-induced increases in oxidative stress or neuroplastic responses to the light environment. Oxidative stress may arise because ALAN suppresses synthesis of the indolamine melatonin, which is synthesized in the brain (and other tissues) and both drives circadian rhythms and acts as a powerful antioxidant [7,11–13]. This can lead to neuron death (zebra finches, *Taeniopygia guttata*) [14] and cognitive impairment (mice, *Mus musculus*) [15]. These effects may occur across tissues [6] or in specific regions [14], so effects on the brain could be holistic or region-specific. Shifts may also occur due to neuroplastic responses when tissue investment shifts in response to environmentally dependent functional demands. For instance, desert ants (*Cataglyphis fortis*) increase investment in visual centres when they move from subterranean nest-tending to above-ground vision-based foraging [16]. Given these scenarios, neuroplasticity may act as part of a feedback loop: if ALAN suppresses melatonin synthesis, investment into the corresponding neural tissues may decrease, resulting in shifts in neural volume [17,18].

**Figure 1.**
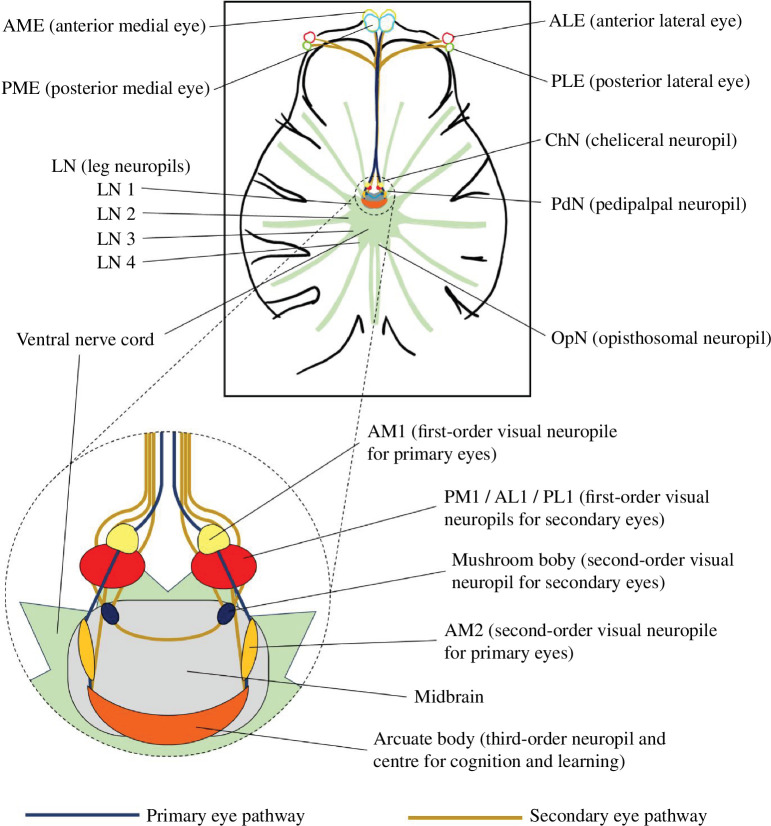
Schematic for the central nervous system (dorsal view) of *H. biapicata*. The top box shows an outline of the entire prosoma, including the pathways of neural tracks extending from the opisthosomal, leg, pedipalpal and cheliceral neuropils, and the black outline shows the positioning of the carapace, chelicerae and coxa of the legs and pedipalps. The dotted circle shows a magnified view of the brains and associated structures, including the primary eye and secondary eye pathways. The paired AM2 neuropils and mushroom bodies sit within the midbrain.

These impacts on the brain have important fitness implications in urban habitats: larger brain volumes and corresponding behavioural flexibility are related to urban success in a range of vertebrates [19–21]. Yet, surprisingly, comparable data for invertebrates—and spiders in particular—are generally lacking. Spiders are ecologically significant terrestrial predators that play key roles in urban ecosystems [22,23]. Like insects, spiders have modular central nervous systems, consisting of anatomically and functionally distinct synaptically dense nerve regions, or neuropils [24], that include two paired visual pathways (figure 1). Anatomical investment in visual processing can vary with ecological need [24,25]. For example, male net-casting spiders (*Deinopis spinosa*) reduce investment in visual processing structures when they mature, because olfactory processing becomes more important for finding mates [26]. There is also increasing evidence that anthropogenic light is attractive to spiders and, in turn, can influence foraging site selection [27,28]. This shift in foraging behaviour can result in net foraging gains [28,29], but it can also negatively affect developmental pathways, resulting in reductions in fitness [8]. Whether any of the observed light-related impacts on spiders are linked to variation in neural structures remains untested.

Here, we used micro-CT imaging to test the effects of short-term exposure to ALAN during late juvenile development, on the volumes of central nervous system structures of sexually mature Australian garden orb-weaving spiders (*Hortophora biapicata*). In this species, ALAN accelerates development to maturity, resulting in shifts in body size and reproductive fitness, and increases juvenile mortality [8], so we predicted exposure to ALAN would increase physiological stress, leading to reduced nervous system volumes. Given the potential for neuroplastic shifts in specific brain structures in responses to the environment, and for oxidative stress to differentially affect tissues, we also predicted that the presence of ALAN would have a greater effect on structures related to vision and melatonin synthesis, such as the primary eye visual neuropils, compared with other structures in the central nervous system [30].

## 2. Method

### (a) Collection, housing and experimental treatments

We collected late juvenile male and female orb-weavers in January 2022 from dark sites (skyglow lux <0.3; Skye Instruments Lux Meter, Llandrindod Wells, Wales, UK) around Melbourne, Australia. Spiders were transported to the laboratory (25°C, 12 h light : 12 h dark lighting cycle) and housed individually in inverted plastic cups (13 cm height, 9 cm diameter). After 3.8 ± 0.2 days, spiders were allocated to either a *Dark at Night* treatment (12 h daytime lighting from a cool-white fluorescent tube, 2000 lux, followed by 12 h natural darkness at night, approximately 0.01 lux scattered light from a cool-white LED; sample sizes: 4 males, 10 females) or a dim *Light at Night* treatment (12 h daytime as above; 12 h ALAN, 20 lux, cool-white LED; 5 males, 10 females). These spiders typically build their webs in open spaces high above the ground and so are likely more exposed to moonlight [31]. While spider age was unknown at the time of collection, the duration of time in the laboratory, final adult body mass and adult prosoma length were comparable for both lighting treatments (electronic supplementary material, table S1). All cups were misted with water three times a week, and spiders were fed with house crickets (*Acheta domesticus*) approximately equal to their own body length twice weekly. We allowed males and females to complete a reproductive event before assay, by mating within their treatment groups. We did this given the potential impact of reproduction on brain volumes [32,33], and the fact that it is ecologically realistic: virginity is a brief and temporary status in invertebrates [34]. Spiders were killed by transferring them to a −20°C freezer for 10 min—males immediately (approx. 1 h) after mating; females were left until they had produced a single egg sac. In total, spiders were exposed to their lighting treatments for 35.40 ± 2.16 days for males (subadult: 10.40 ± 2.22; adult: 25.00 ± 2.33) and 49.15 ± 1.92 days for females (subadult: 9.45 ± 1.35; adult: 39.70 ± 1.91). Adult prosoma length, an index of body size, was measured from micro-CT scans (see below) using ImageJ [35].

### (b) Tissue staining

After freezing, we immediately transferred the spider to chilled 70% ethanol and removed the legs (broken off at the coxa), pedipalps, chelicerae and opisthosoma with fine forceps. The prosoma was then stored in 70% ethanol prior to tissue analysis. Legs were stored separately in 70% ethanol, and the opisthosoma was frozen at −80°C for separate analyses. Tissue staining methods were adapted from [36]. Each sample was passed through an ethanol dehydration series (70%–80%–85%–90%–96%–99% ethanol in water, remaining in each concentration for 20 min). After dehydration, we placed each sample in an Eppendorf tube containing 1% phosphotungstic acid (PTA) in 70% ethanol. Samples remained in the PTA stain for approximately 60 days until the stain had completely penetrated the prosoma, as visualized through a preview using a Phoenix Nanotom M nanoCT machine (Waygate Technologies). To increase stain penetration, we replaced the stain in each Eppendorf tube every 2–4 days and applied a vacuum to the tubes (with lids open) for 3 min (then left for 10 min before releasing the vacuum) three times per week.

### (c) Micro-CT settings

Samples were scanned at the University of Melbourne using a Phoenix Nanotom M nano-CT operated using xs control and phoenix datos|x acquisition software (Baker Hughes Digital Solutions GmbH). Prosomas were held in 0.2–0.5 ml PCR tubes that were mounted using a hot glue gun onto glass rods for scanning. Prosomas were scanned at a coarser resolution (1199 projections), while focused scans on brains required higher resolution scanning (1800 projections and 2 images were averaged per projection) (scan parameters are summarized in electronic supplementary material, table S2). Samples were rotated through a full 360° (electronic supplementary material, table S2). The focused scan resolution varied between specimens used for analyses (pixel size: 2.3–2.9 μm) due to the optimization of settings, but did not differ between groups (*F*_3,25_ = 0.64, *p* = 0.59). Additionally, there was no relationship between pixel size and the calculated volume of AM1 neuropils (*F*_1,27_ = 0.012, *p* = 0.91).

Volume reconstruction of the micro-CT data was performed using the phoenix datos|x reconstruction software applying an inline median filter and ROI-CT filter during reconstruction. Higher resolution scans were collected using a finer focal mode and reconstructions performed with and without 2 × 2 software binning (sfb—mode 1, electronic supplementary material, table S2). Data were imported to Avizo (Thermo Fisher Scientific) to reorient specimens so that the anterior medial eyes were oriented upward through a dorsal plane of the prosoma.

### (d) Central nervous system segmentation

Segmentations were performed manually in ITK-SNAP [37], following a similar approach to that of previous studies [18,36]. For all females (*n* = 10 *Dark at Night*, 10 *Light at Night*) and males (*n* = 4 *Dark at Night*, 5 *Light at Night*), volumes of the midbrain, whole ventral nerve cord (VNC), pedipalpal neuropils (PdN) and cheliceral neuropils (ChN) were measured using the coarser resolution full prosoma scans (electronic supplementary material, table S2). Mushroom bodies (MB), arcuate body (AB), first-order primary eye neuropils (AM1), second-order primary eye neuropils (AM2) and first-order secondary eye neuropils (PM1/AL1/PL1) were measured using the higher resolution focused brain scans (electronic supplementary material, table S2). Structure boundaries were identified based on tissue contrast, observable neural pathways and reference to previous literature [24,36] (electronic supplementary material, figure S1). All analyses were performed blind to the identity of each sample.

### (e) Statistical analysis

Statistical analyses were performed in R v. 4.2.2 [38]. Data are available on Dryad [39]. Prior to analyses, data were transformed to ensure normality of residuals, where required, using exponent optimization. To assess the effects of lighting treatment on neural pathways and the relationships between neuropils, we used principal components analysis (PCA) to summarize variation among the neuropil groups. We included the volumes (relative to prosoma length to account for body size) of six neuropil types: (i) first-order primary eye neuropils (AM1); (ii) second-order primary eye neuropils (AM2); (iii) first-order secondary eye neuropils (PM1/AL1/PL1); (iv) mushroom bodies (MB); (v) arcuate body (AB); and (vi) the remainder of the central nervous system (ventral nerve cord + midbrain + PdN + ChN). The PCA used scaled and centred variables, and returned three axes of variation (principal components; PCs) with an eigenvalue >1.0. These PCs were then used as response variables in separate linear regressions. Each model included lighting treatment, sex and their interaction. Although days in the laboratory covaried with sex (as females were permitted additional time to produce an egg sac), it did not differ between lighting treatments (electronic supplementary material, table S1) and so was excluded from the models. Thus, although sex is included in the models, it reflects both sex differences and duration of exposure to laboratory conditions and treatment, so we cannot infer relationships between sex and brain volumes directly.

## 3. Results

### (a) Effects of artificial light at night on neural pathways

The PCA on relative neuropil volumes produced three axes of variation (PCs) with eigenvalues >1.0, together explaining 71.5% of the measured variation (table 1). PC1 was positively loaded by the secondary eye visual pathway (PM1/AL1/PL1 and MB) and by the rest of the central nervous system. PC2 was positively loaded by the primary visual pathway (AM1 and AM2) and the AB. PC3 was negatively loaded by AM2 and positively loaded by MB and AB.

**Table 1.** Principal components for central nervous system structure volumes. Loadings in bold strongly correlate (>0.4) with their corresponding principal component.

	PC1	PC2	PC3
principal component parameters	secondary visual pathway and rest of central nervous system	primary eyes visual pathway	male-biased (AB+MB) versus female-based (AM2) structures
eigenvalue	1.87	1.24	1.18
proportion of variance	31.1%	20.7%	19.7%
**structure loadings**			
paired first order visual neuropils for the anterior median eyes (AM1)	−0.20	**0.67**	−0.38
paired second order visual neuropils for the anterior median eyes (AM2)	0.27	**0.41**	**−0.46**
first order visual neuropils for the secondary eyes (PM1/AL1/PL1)	**0.60**	−0.07	−0.17
mushroom bodies	**0.48**	0.14	**0.48**
arcuate body	0.10	**0.59**	**0.57**
remainder of central nervous system (VNC, midbrain, ChN, PdN)	**0.53**	−0.11	−0.25

We found no significant interaction effects between lighting treatment and sex for any PC. There was no effect of lighting treatment on PC1, and weak evidence for an effect of sex, suggesting that volumes in the secondary eye visual pathway may be larger in females than males (table 2*a*). For PC2, which describes the primary eye visual pathway and the arcuate body (where the pathway terminates), there were significant effects of both lighting treatment and sex: structure volumes were smaller for *Light at Night* spiders compared with the *Dark at Night* control group, and smaller for females compared with males (table 2*b*). Finally, for PC3, there was no effect of lighting treatment, but a significant positive effect of sex, indicating that mushroom bodies and arcuate bodies together tended to be bigger in males, while the second-order primary eye visual neuropils tended to be larger in females (table 2*c*).

**Table 2.** Linear regression models for the effects of lighting treatment, sex and their interaction on the first three principal components explaining variation in central nervous system volumes.

predictors	estimate ± s.e.	statistic	*p*‐value
*(a) PC1: secondary visual pathway and rest of central nervous system*			
lighting treatment	−0.33 ± 0.60	*F*_1,25_ = 0.02	0.89
sex	−1.46 ± 0.79	*F*_1,25_ = 3.45	0.08
lighting × sex	0.85 ± 1.08	*F*_1,25_ = 0.63	0.44
*(b) PC2: primary eyes visual pathway*			
lighting treatment	−1.30 ± 0.40	*F*_1,25_ = 10.07	**0.004**
sex	0.59 ± 0.53	*F*_1,25_ = 8.05	**0.009**
lighting × sex	0.80 ± 0.72	*F*_1,25_ = 1.23	0.28
*(c) PC3: male-biased (AB+MB) versus female-based (AM2) structures*			
lighting treatment	0.10 ± 0.42	*F*_1,25_ = 0.23	0.63
sex	1.18 ± 0.56	*F*_1,25_ = 11.63	**0.002**
lighting × sex	0.21 ± 0.76	*F*_1,25_ = 0.08	0.78

## 4. Discussion

We demonstrate, for the first time, the effect of ALAN on the brain structure of an invertebrate. We found that exposure to ALAN from the late juvenile through to the early adult phase of the spider lifecycle resulted in a marked reduction in neuropil volumes. Moreover, these effects were strongest for the first-order visual neuropils of the primary eyes suggesting the impacts of ALAN were most pronounced in the primary visual pathway. Here, we discuss how this outcome could potentially be driven either by oxidative stress or by a neuroplastic response to hormone synthesis and the light environment.

We observed an ALAN-related volume reduction in the primary visual pathway and the first-order visual neuropils in particular. This is the first demonstration of such an effect of ALAN on an invertebrate. Previous studies in vertebrates have found impaired cognition and neural death associated with increased oxidative stress when exposed to ALAN [14,15]. If reduced neuropil volumes were driven by oxidative stress—the effects of which are often evident across tissue types [6]—we might expect effects across all neuropils. Hence, the lack of an overall decrease in central nervous system volumes in our spiders may suggest that oxidative stress is a weaker explanation for the results observed. However, oxidative stress has been associated with region-specific neuron death in zebra finches [14], so it remains possible that first-order visual neuropils are more susceptible to oxidative stress, leading to the structure-specific volume reductions we observed. Given the relatively short exposure period in our experiment, the consequences of ALAN-induced oxidative stress may become more evident over extended exposure periods [8,40,41]. Additionally, given that our *Dark at Night* treatment (approx. 0.01 lux) was not complete darkness, the effects of ALAN exposure may be stronger in comparison to spiders reared under complete darkness.

Our result could be explained as a neuroplastic response to ALAN. Across taxa, investment in visual processing centres depends on the light environment and associated functional needs. For example, desert ants (*Cataglyphis fortis*) can show neuroplastic shifts in the visual centres when they move from subterranean nest-tending to above-ground vision-based foraging [16], whereas male net-casting spiders (*Deinopis spinosa*) reduce investment in visual processing when they mature, because olfactory processing becomes more important for finding mates [26]. Given that *H. biapicata* is a non-visual predator, increased oxidative stress due to ALAN may have stimulated the observed reduction in visual system investment. This should be further explored in the future by comparison with other investments such as reproductive output, to determine if ALAN is driving energetic trade-offs. Decreases in neuropil volumes may also reflect decreases in neuroendocrine activity. Based on PC loadings, we found a stronger reduction in the first-order primary eye visual neuropils (AM1). In spiders, the AM1 neuropils are associated with NS A and B cells, which are likely candidates for the production of melatonin [30]. Reduced neural activity can lead to reduced brain volumes [42–44], so if ALAN suppresses melatonin activity and hence activity in this region of the brain, the volume of first-order primary eye visual neuropils may be further reduced. Circadian entrainment occurs through the secondary, but not primary, eyes of jumping spiders [45] and wolf spiders [46], although orb-weaver primary eyes do show circadian oscillations in sensitivity [47], so these processes may differ between taxa. It is important to also consider the potential effects of sex, as we found differences in neuroanatomy between males and females. Our experimental design prevents us from disentangling the relative importance of sex differences, exposure duration and reproductive events. However, males and females have different ecological demands [48,49], and sex-specific effects of ALAN—specifically with respect to melatonin—are common [14,50,51], warranting further investigation.

Our results have important implications for urban exploiting species. Variation in brain volumes can be an important predictor of urban success: larger brains facilitate effective behavioural responses to novel conditions and problems [19–21]. However, once animals are established in cities, pollutants such as ALAN may negatively impact the traits that facilitate their success. Our results suggest that the brains of spiders show neuroplastic responses to the light environment, potentially as a result of oxidative stress or changes in neural activity. In these orb-weavers, the particular effects on the primary visual pathway, where melatonin is potentially synthesized, may explain previously observed fitness effects, such as shifts in development rate, increases in mortality and changes in foraging [8,28]. It will be important to further investigate the potential fitness effects of these neural shifts. Furthermore, urban animals experience a multitude of stressors, which could exacerbate the impacts of ALAN, particularly if longer ALAN exposure or more stressful conditions result in increased oxidative stress. As improving technology allows greater insight into neural structure and function, the study of brains may provide a key link between the natural biology of a species and their responses to an increasingly illuminated world.

## Data Availability

Data are available on Dryad [39]. Supplementary material is avaliable online [52].
